# EXTERNAL HOME DISREPAIR ASSOCIATED WITH OBJECTIVE SLEEP DISTURBANCES IN LOW-INCOME OLDER ADULTS WITH DISABILITIES

**DOI:** 10.1093/geroni/igz038.3358

**Published:** 2019-11-08

**Authors:** Safiyyah M Okoye, Nancy Perrin, Sarah Szanton, Adam P Spira

**Affiliations:** 1 Johns Hopkins University Bloomberg School of Public Health, Baltimore, Maryland, United States; 2 Johns Hopkins University, Baltimore, Maryland, United States; 3 Johns Hopkins School of Nursing, Baltimore, Maryland, United States; 4 Johns Hopkins Bloomberg School of Public Health, Baltimore, Maryland, United States

## Abstract

Sleep disturbances are linked to poor health, loss of independence and mortality in older adults. Rates of poor sleep are higher among socioeconomically disadvantaged older adults. Understanding how environmental factors may affect sleep in this population could lead to interventions to improve sleep-related health outcomes. We determined cross-sectional associations of home and neighborhood conditions with sleep parameters, measured by wrist actigraphy, in 136 low-income, predominantly African-American older adults with disabilities. Primary predictors were third-party-rated objective indicators of disrepair or disorder based on: 1) inside-home conditions (e.g., evidence of pests, tripping hazards, clutter); 2) outside-home conditions (e.g., broken windows, crumbling foundation); and 3) neighborhood conditions (e.g., litter, graffiti, vacant buildings). Outcomes were actigraphic total sleep time (TST; total number of minutes in bed spent asleep), wake time after sleep onset (WASO; total number of minutes spent awake after initially falling asleep), and sleep efficiency (SE; % of time in bed spent asleep). Presence of one or more outside-home conditions indicating disrepair or disorder was associated with 36.3-minutes shorter TST, 18.1-minutes more WASO, and 4.7% lower SE (all p <0.05). Conditions inside the home and of the neighborhood were not associated with sleep. These preliminary findings suggest that among low-income older adults with disabilities, external-home disrepair is associated with objectively measured WASO, TST, and SE. External-home disrepair may affect sleep through physical, psychosocial and behavioral pathways. Further research should examine longitudinal associations between external-home conditions and objectively measured sleep in socioeconomically disadvantaged older adults.

